# Mr. Key's Case of Ulceration of the Cartilages of the Larynx

**Published:** 1835-04-01

**Authors:** J.H. Freeman

**Affiliations:** Guy's Hospital


*To the Editor of the Medical Quarterly Review.*

SIR: I beg leave to enclose you some notes of three cases which occurred in the wards of Guy's Hospital, under the care of Mr. Key.

If you should deem them worthy of a place in the next Number of your Review, I have Mr. Key's permission to make that use of them.

I am, sir, your obedient servant,

J. H. FREEMAN.

*Guy's Hospital; March* 20, 1835.

### I. *Ossification and Ulceration of the Cartilages of the Larynx, with Fistulous Opening.*

George Blackgrove, æt. forty-eight. During the former part of his life he was employed in some iron-mills, and was accustomed to drink spirits freely; but his habits lately have been temperate. He received a severe burn on the head five years ago, which healed very slowly (in twelve months), and reduced him considerably.

After that, his occupation was changed, and he worked in a tan-yard, where he was much exposed to damp and cold: the early consequence was an abscess which formed behind the left ear, and proved as troublesome as the burn: after having been a patient at several public institutions, he was at length cured in fifteen months. For the last four or five weeks, his health has not been so good as usual; and, fourteen days ago, he had a small swelling, attended with pain, on the left side of the larynx, followed successively by two on the opposite side: they all burst, and discharged much. Four days before his admission, he noticed a hissing sound proceeding from the ulcer on the left side, and he coughed almost incessantly for about two days: at last he applied for relief at the hospital as an out-patient. He was admitted to the house the following day, January 3d; and, on examination, a large portion of the cricoid cartilage on the left side, and of the thyroid on the right, was found ossified and denuded: there was a fistulous opening through the crico-thyroid membrane, through which air passed very freely while speaking, and generally without giving pain. He states that six months ago he strained himself while carrying a heavy weight, and that his throat has always been uneasy since.

Local applications, and those of a very mild nature only, were applied for several days; on the 7th January, Mr. Key ordered him Iodinæ gr. ss.; Potass. Hydriod. gr. iij.; Syr. Papav. ʒss.

10th. His appetite, which was deficient when he was admitted, is increased; the ulcers somewhat improved, appearing less inclined to extend.—Pergat.

12th. Mr. Key attempted to remove a portion of the cricoid cartilage, but found it firm.

16th. His health and appearance are both much better: he requested a more plentiful diet, which was allowed him. A small portion of the cricoid cartilage was removed.

20th. He was ordered, as a gentle stimulant, a lotion of very dilute nitric acid (gtt. ij. ad ℥j. aq.); but, by mistake, he applied some pure acid, which was in the ward. This destroyed the integuments to some extent, but does not appear to have affected the cartilage.

21st. The ulcer is much inflamed. No ill effect followed the application of the acid except the extension of the ulcerating surface. Bread poultices were applied, and the iodine continued.

23d. His throat is much better; the cartilage appears quite firm, and the ulcers on the right side cicatrizing.

28th. The smaller ulcer on the right side healed. One bare spot of cartilage can be felt by the probe, in the larger one on the same side. The opening in the larynx on the left side smaller.—Pergat.

The opening was closed on the 12th of February, and he left the hospital quite well about three weeks afterwards. He suffered no inconvenience from the iodine.

